# Apoptotic DNase network: Mutual induction and cooperation among apoptotic endonucleases

**DOI:** 10.1111/jcmm.16665

**Published:** 2021-06-04

**Authors:** Alexei G. Basnakian, Christopher L. Moore

**Affiliations:** ^1^ University of Arkansas for Medical Sciences Little Rock AR USA; ^2^ Central Arkansas Veterans Healthcare System Little Rock AR USA

**Keywords:** apoptosis, cell death, DNA fragmentation, DNase, endonuclease, network

## Abstract

DNA fragmentation produced by apoptotic DNases (endonucleases) leads to irreversible cell death. Although apoptotic DNases are simultaneously induced following toxic/oxidative cell injury and/or failed DNA repair, the study of DNases in apoptosis has generally been reductionist in approach, focusing on individual DNases rather than their possible cooperativity. Coordinated induction of DNases would require a mechanism of communication; however, mutual DNase induction or activation of DNases by enzymatic or non‐enzymatic mechanisms is not currently recognized. The evidence presented in this review suggests apoptotic DNases operate in a network in which members induce each other through the DNA breaks they produce. With DNA breaks being a common communicator among DNases, it would be logical to propose that DNA breaks from other sources such as oxidative DNA damage or actions of DNA repair endonucleases and DNA topoisomerases may also serve as triggers for a cooperative DNase feedback loop leading to elevated DNA fragmentation and subsequent cell death. Therefore, mutual induction of apoptotic DNases has serious implications for studies focused on activation or inhibition of specific DNases as a strategy for therapeutic intervention aimed at modulation of cell death.

## 1 DISCOVERY OF APOPTOTIC DNASES

Apoptotic DNases are endonucleases responsible for fragmentation of DNA in apoptosis and other types of cell death.^1^ Initial studies observed delayed changes in DNA following irradiation cell injury, but preceding cell death, might be the result of ‘enzyme activation’.^2^ Non‐necrotic cell death associated with normal physiological involution or triggered by noxious agents was recognized as programmed cell death and defined as apoptosis in the seminal work on apoptosis by Kerr, Wyllie and Currie in 1972.^3^ The importance of DNases to DNA fragmentation associated with cell injury and death was revealed by Skalka et al in 1976, who demonstrated irradiation of lymphocytes in vivo caused post‐irradiation internucleosomal fragmentation of DNA.^4^ The study established two very important facts. First, DNA degrades into regular fragments after irradiation. Second, the DNA fragments could not be distinguished from fragments produced by endonuclease digestion. This study confirmed post‐irradiation DNA damage is enzymatically mediated and demonstrated internucleosomal DNA fragmentation occurs in vivo. A later study by Wyllie^5^ associated the enzymatic digestion pattern with apoptosis in glucocorticoid‐induced cell death of lymphoid cells in vitro. Rat thymocytes treated with methylprednisolone produced an internucleosomal DNA fragmentation pattern similar to isolated nuclei exposed to endonuclease digestion. Additionally, the observed morphological chromatin condensation of apoptosis correlated closely with the products of DNA digestion. The DNase involved in early studies of chromatin structure that produced internucleosomal DNA fragmentation was initially called ‘Ca/Mg‐dependent endonuclease’ due to its cation requirements.^6^ This activity could be identified by the production of a 200‐bp‐ladder pattern in agarose gel electrophoresis of DNA from isolated cell nuclei incubated in the presence of Ca^2+^ and Mg^2+^ ions.^7^ Wyllie suggested apoptosis may be induced by activation of this Ca/Mg‐dependent endonuclease.^5^ However, the studies of the next two decades did not confirm the existence of a single apoptotic endonuclease, but instead showed that nine DNases acting together perform apoptotic DNA fragmentation. This group of the endonucleases included the venerable deoxyribonuclease 1 (DNase I), well known since the 1940s,^8^ three DNase I homologs, DNase 2 with its two homologs, endonuclease G (EndoG) and caspase‐activated DNase (CAD). These DNases are covered in greater detail in the review by Keyel.^9^

## 2 EVIDENCE OF AN APOPTOTIC DNASE NETWORK

When studied individually by different research groups, each of the DNases behaved as the central regulator of all apoptotic DNA fragmentation because its overexpression induced apoptosis and its genetic inactivation inhibited DNA fragmentation–associated cell death in different models.^10^, ^11^, ^12^, ^13^ However, this ‘necessary and sufficient’ character seems to be shared by different DNases even within the same experimental model. For example, graphene cytotoxicity in vitro and cisplatin nephrotoxicity in vivo were shown to be alleviated by inhibition of either DNase I or EndoG.^14^, ^15^ Three enzymes, DNase I, DNase γ (DNase1L3) and CAD, were shown to be important for γ irradiation–induced cell death in vivo.^16^, ^17^ Both EndoG and CAD were shown to be necessary for cardiac hypertrophy,^18^, ^19^ while both DNase I and DNase γ were shown to be crucial for acetaminophen‐induced acute liver injury.^17^, ^20^ In the latter model,^20^ DNase I knockout mice were protected against drug‐induced liver injury; however, the protection of DNase I knockouts was an apparent off‐target effect because the most active and abundant endonuclease expressed in liver is DNase γ.^21^

While individual DNases can appear to be necessary and sufficient for DNA fragmentation leading to cell death, even when tested within the same model, temporal relationships in DNase induction suggested a cooperative relationship. Follow‐up studies demonstrated that in some models, the induction of different apoptotic DNases occurred at different time‐points after a cell death stimulus. Apoptotic DNA fragmentation induced by the protein kinase inhibitor staurosporine in proliferating N1E‐115 neuroblastoma cells was associated with induction of CAD followed by its disappearance that coincided with the induction of DNase γ.^22^ In kidney ischaemia‐reperfusion in rats and mice, DNase I was induced with a peak at 16 hours during the reperfusion stage, while EndoG was induced only when DNase I went down several hours later.^23^ In both of these models, DNases appear to have a way of signalling and regulating the expression of each other. In acute kidney injury induced by cisplatin, EndoG could be induced only in DNase I–expressing wild‐type mice, while DNase I knockouts did not have EndoG induction in the kidney.^15^ Again, this clearly suggested a signalling link between the two enzymes.

The notion of a cooperative relationship among DNases is reinforced by two recent studies that demonstrated both EndoG and DNase I acting alone can induce each other and several other apoptotic DNases.^24^, ^25^ In the first study, an overexpression of mature EndoG in kidney tubular epithelial NRK‐52E cells was shown to increase expression of CAD and four endonucleases including DNase I and its three homologs, DNase X (DNase1L1), DNase1L2 and DNase γ (DNase1L3).^25^ The induction of the DNase I–type endonucleases was associated with DNA degradation in promoter/exon 1 regions of the endonuclease genes. These results, together with findings on colocalization of immunostained endonucleases and TUNEL positivity for DNA fragmentation, suggested that DNA fragmentation after EndoG overexpression was caused by DNase I–like endonucleases and CAD in combination with the inductive endonuclease, EndoG. In the second study,^24^ NRK‐52E cells were transfected with the DNase I gene or its inactive mutant in a pECFP expression vector, while control cells were transfected with the empty vector. This study showed that DNase I, but not its inactive mutant, induced all other apoptotic DNases at varying time periods after transfection. Similar to EndoG, overexpression of DNase I caused DNA breaks in promoter/exon 1 regions of several apoptotic DNase genes and elevated protein expression of several DNases. The mechanism of this is not very obvious; however, even a single‐stranded DNA break (nick) would cause a dramatic decrease of DNA supercoiling, which, in turn, may affect DNA exposure to proteins participating in transcription. In particular, increased DNA binding by RNA polymerase or decreased binding of repressors would result in elevation of transcription. It is interesting that the DNase promoter not cleaved by DNase I was the one of EndoG. The likely reason for this is DNase I has preferential specificity to AT‐rich sequences,^26^ like those found in promoter TATA boxes.^27^ On the other hand, the entire EndoG gene, including its promoter, is highly GC‐rich and presents as one large CpG island,^28^ which would be relatively resistant to DNase I cleavage. The question remains, why is DNase I able to induce EndoG? It may be DNase I scissions are located outside of EndoG gene. These reports were the first evidence that endonucleases may be induced by the DNA‐degrading activity of DNase I.

Mutual induction by directly targeting promoter regions of other DNases is not the only mean by which DNases can cooperate. In terms of sequence specificity, DNases often overlap with each other. However, due to some specificity of the initial cleavage, endonucleases may cooperate with each other in a manner where the product of one may act as the substrate of another. In particular, DNase I was shown to stimulate the ability of human recombinant EndoG to produce double‐stranded DNA breaks in naked DNA and chromatin in vitro.^29^ Cooperative activity between CAD and DNase γ resulting in internucleosomal DNA fragmentation was reported in tumour necrosis factor–induced apoptosis in HT‐29 cells.^30^

## 3 OTHER CONTRIBUTORS TO THE DNASE NETWORK

**FIGURE 1:**
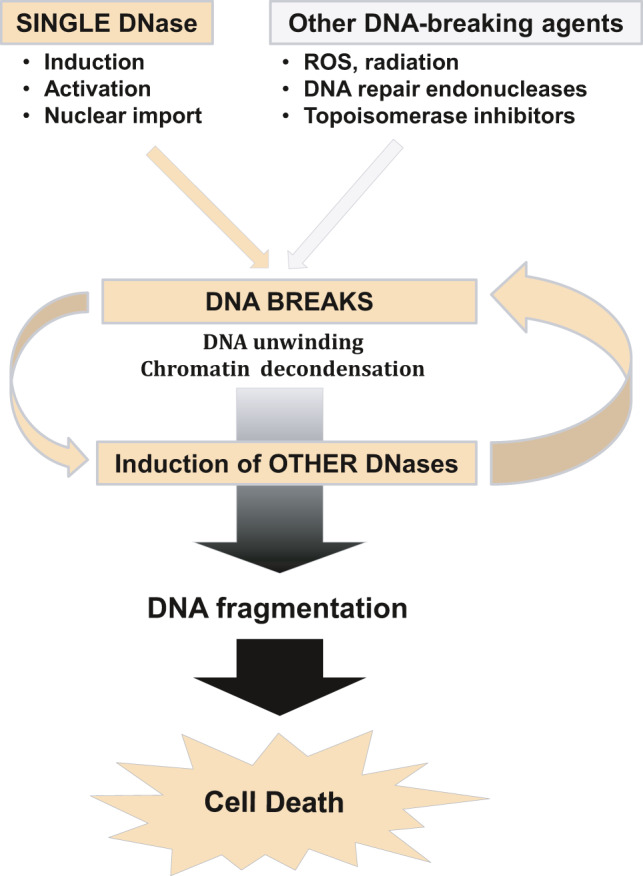
Hypothetical scheme of the vicious cycle of apoptotic DNases acting as a network. This cycle can be activated by a single activated or induced apoptotic DNase, AP endonuclease, ROS/radiation or topoisomerase inhibitor

DNases are just one source of DNA breaks, and it is logical to propose that DNA breaking mechanisms in general, including radiation, mitochondria‐ and drug‐induced reactive oxygen species (ROS), apurinic/apyrimidinic (AP) DNA repair endonucleases and DNA topoisomerases (stopped mid‐reaction by topoisomerase inhibitors), may contribute to apoptotic DNase induction through a feedback loop or act cooperatively with apoptotic DNases (Figure 1). There is overwhelming collective evidence that ROS induce direct DNA breaks as well as DNA modifications, for example 8‐hydroxyguanosine requiring DNA repair with participation of AP‐endonucleases. This entire process is associated with DNA breaks and activation of DNA damage pathways and apoptosis, indirectly leading to enzymatic DNA fragmentation. Similarly, topoisomerase inhibitors induce protein‐bound DNA breaks and activate DNA damage pathways and apoptosis with its own DNase‐mediated DNA fragmentation.^31^, ^32^ Human AP endonuclease Ape 1 and its N‐terminally truncated form were shown to participate in apoptotic DNA degradation and potentially cooperate with CAD.^33^ CRN‐1, a *C* *elegans* homologue of human AP endonuclease FEN‐1 that is normally involved in DNA replication and repair, was reported to cooperate with *C elegans* Endo G (CPS‐6) in DNA fragmentation, utilizing the endonuclease activity of CPS‐6 and both the 5’‐3’ exonuclease activity and gap‐dependent endonuclease activity of CRN‐1.^34^

Whether initial oxidative DNA breaks, DNA repair endonuclease‐mediated DNA breaks or topoisomerase‐bound DNA breaks directly contribute to the pool of breaks and directly activate or cooperate with apoptotic endonucleases has not been studied. The absence of research on such relationships could be because the interactions are difficult to distinguish and study separately due to lack of appropriate research tools or an apparent lack of interest. However, even in a simple in vitro model, the anticancer drug bleomycin able to directly induce DNA strand breaks, induced transcription of DNase I and EndoG genes inserted in an expression vector as effectively as recombinant endonucleases.^24^, ^25^ Placing DNA breaks at the centre of communication between all DNA‐damaging agents makes it easy to imagine that a single endonuclease activated by an external stimulus, extracellular or intracellular ROS, or DNA proliferation and repair agent could trigger a vicious cycle of apoptotic DNases to destroy host cell DNA and induce cell death (Figure 1).

## 4 CONCLUSION

The precise mechanisms that enable apoptotic DNases to act simultaneously during cell death remain unclear. Central questions include how DNases mutually induce each other and how non‐enzymatic mechanisms, such as ROS, radiation or drug‐induced DNA damage, activate DNases. In light of the evidence for an apoptotic DNase network, studies involving DNases need to consider DNases do not act independently from each other. Studies designed to activate a single DNase in an attempt to stimulate cell death (eg targeting cancer cells) or inactivate a particular DNase for the purpose of tissue protection from an injury would require monitoring the activity of other DNases. Perhaps, a better strategy for tissue protection might be simultaneous inhibition of several DNases by broad‐spectrum inhibitors instead of a single highly specific inhibitor.

## CONFLICT OF INTEREST

The authors declare no competing financial interests.

## AUTHOR CONTRIBUTION

**Alexei G. Basnakian:** Conceptualization (equal); Data curation (equal); Investigation (equal); Project administration (equal); Writing‐original draft (equal); Writing‐review & editing (equal). **Christopher L. Moore:** Conceptualization (equal); Formal analysis (equal); Writing‐review & editing (equal).

## Data Availability

The data that support the findings of this study are available from the corresponding author upon reasonable request.
